# CBFβ-MYH11 interferes with megakaryocyte differentiation via modulating a gene program that includes GATA2 and KLF1

**DOI:** 10.1038/s41408-019-0194-8

**Published:** 2019-03-08

**Authors:** Guoqiang Yi, Amit Mandoli, Laura Jussen, Esther Tijchon, Maaike G. J. M. van Bergen, Gaëlle Cordonnier, Marten Hansen, Bowon Kim, Luan N. Nguyen, Pascal W. T. C. Jansen, Michiel Vermeulen, Bert van der Reijden, Emile van den Akker, Jonathan Bond, Joost H. A. Martens

**Affiliations:** 1 Department of Molecular Biology, Faculty of Science, Radboud Institute for Molecular Life Sciences, Radboud University Nijmegen, 6525 GA Nijmegen, The Netherlands; 2 Department of Laboratory Medicine, Laboratory of Hematology, Radboud University Medical Center, Radboud Institute for Molecular Life Sciences, Nijmegen, The Netherlands; 3 Université Paris Descartes Sorbonne Cité, Institut Necker-Enfants Malades (INEM), Institut national de recherche médicale (INSERM) U1151; and Laboratory of Onco-Hematology, Assistance Publique-Hôpitaux de Paris (AP-HP), Hôpital Necker Enfants-Malades, Paris, France; 4 Department of Hematopoiesis, Sanquin Research, and Landsteiner Laboratory, Academic Medical Center, University of Amsterdam, Amsterdam, 1066 CX The Netherlands; 5 Department of Molecular Biology, Faculty of Science, Radboud Institute for Molecular Life Sciences, Oncode Institute, Radboud University Nijmegen, 6525 GA Nijmegen, The Netherlands; 6 Systems Biology Ireland, School of Medicine, University College Dublin, Dublin, Ireland; 7 National Children’s Research Centre, Our Lady’s Children’s Hospital Crumlin, Dublin, Ireland; 8 Dipartimento di Biochimica, Biofisica e Patologia generale, Università degli Studi della Campania ‘Luigi Vanvitelli’, Vico L. De Crecchio 7, 80138 Napoli, Italy

## Abstract

The inv(16) acute myeloid leukemia-associated CBFβ-MYH11 fusion is proposed to block normal myeloid differentiation, but whether this subtype of leukemia cells is poised for a unique cell lineage remains unclear. Here, we surveyed the functional consequences of *CBFβ-MYH11* in primary inv(16) patient blasts, upon expression during hematopoietic differentiation in vitro and upon knockdown in cell lines by multi-omics profiling. Our results reveal that primary inv(16) AML cells share common transcriptomic signatures and epigenetic determiners with megakaryocytes and erythrocytes. Using in vitro differentiation systems, we reveal that CBFβ-MYH11 knockdown interferes with normal megakaryocyte maturation. Two pivotal regulators, GATA2 and KLF1, are identified to complementally occupy RUNX1-binding sites upon fusion protein knockdown, and overexpression of GATA2 partly induces a gene program involved in megakaryocyte-directed differentiation. Together, our findings suggest that in inv(16) leukemia, the CBFβ-MYH11 fusion inhibits primed megakaryopoiesis by attenuating expression of GATA2/KLF1 and interfering with a balanced transcriptional program involving these two factors.

## Introduction

Core-binding transcription factors (CBFs) have been proposed to shape both stem cell self-renewal and differentiation, and their dysfunction could potentially lead to cancer pathogenesis^1^. The CBFs are heterodimeric complexes composed of two distinct subunits, alpha and beta^2^. The CBF α-subunit is encoded by the RUNX family (usually RUNX1/AML1 in the hematopoietic cells) and directly contacts the DNA sequence, whereas the non-DNA-binding CBF β-subunit is thought to facilitate stabilizing the DNA affinity of the CBF complex. CBFs are often mutated in acute myeloid leukemia (AML), for example, in t(8;21) AMLs, characterized by expression of the *AML1-ETO* fusion gene, or inv(16) AMLs, delineated by the presence of the *CBFβ-MYH11* (CM) event^3^. *CBFβ-MYH11* encodes a fusion protein between CBFβ and smooth muscle myosin heavy chain (SMMHC/MYH11), and is associated with AML FAB subtype M4Eo accounting for around 6% of AML cases^4–6^. However, our understanding of its roles in leukemogenesis remains incomplete.

Expression of CBFβ-MYH11 is able to disrupt normal myeloid differentiation, predispose for AML initiation, and cause full leukemia transformation upon the acquisition of additional genetic changes^7,8^. A recent study revealed that CBFβ-MYH11 maintains inv(16) leukemia by obstructing RUNX1-mediated repression of MYC expression, which is featured by the replacement of SWI/SNF for PRC1 at MYC distal enhancers^9^. However, at which differentiation stage CBFβ-MYH11 blocks myeloid differentiation is still unclear. Mutational analysis of FACS-purified hematopoietic stem cells (HSCs) as compared to leukemia cells confirmed the presence of CBFβ-MYH11 in HSCs, suggesting that the fusion event is involved in setting up a preleukemic cell state^10^. Further pursuing which differentiation pathway exactly is targeted by the oncoprotein would be needed.

At the molecular level, CBFβ-MYH11 in a complex with RUNX1 acts as a transcriptional regulator, which can depending on local genomic context, activate and repress genes involved in self-renewal, differentiation, and ribosomal biogenesis^6,11,12^. Our previous findings have shown that a variety of cell surface markers increase in expression levels upon knockdown of CBFβ-MYH11 in the inv(16) cells, including those for the monocytic and megakaryocytic lineages^11^. In addition, mouse studies revealed that expression of the CBFβ-MYH11 protein causes abnormal erythropoiesis and gives rise to preleukemic pre-megakaryocyte/erythrocyte progenitors^8,13^. Overall, these results potentially implicate a role of the CBFβ-MYH11 fusion in skewing cell differentiation orientation.

To investigate whether *CBFβ-MYH11* specifically blocks megakaryocyte/erythrocyte differentiation in the context of human hematopoiesis, and further probe its molecular mechanisms, we analyzed multiple transcriptomic and epigenomic profiles of inv(16) AMLs, several normal hematopoietic cell types and in vitro single-oncogene models. Our findings reveal a clustering of inv(16) AMLs towards megakaryocytes and erythrocytes based on DNA accessibility and H3K27ac-based super-enhancer (SE) profiles. Further molecular exploration indicates that CBFβ-MYH11 seems to be involved in interfering with normal differentiation through transcription deregulation and occupancy replacement of the transcription factors GATA2 and KLF1. Together, these results suggest that controlled expression of KLF1 and GATA2 expression is essential for inv(16) AML development.

## Materials and methods

### Human cells collection and sequencing

Leukemic samples were either obtained from bone marrow or peripheral blood for subsequent processing. Patients cells and cell lines were processed through multiple steps as previously reported^11^, and then subjected to high-throughput transcriptome and chromatin immunoprecipitation (ChIP) sequencing for histone marks, CBFβ-MYH11 fusion, RUNX1, and GATA2 as described in the Supplementary Information.

### Assays

Cell culture, flow cytometry, cytospin, differentiation of iPSCs towards the granulocytic lineage, nuclear extraction preparation, pulldown, and mass spectrometry analysis were performed as detailed in the Supplementary Information.

### Bioinformatics analysis

#### Peak calling

After read mapping to the hg19 reference genome using BWA^14^ and removal of PCR duplicates by Picard *MarkDuplicates* option (http://broadinstitute.github.io/picard/), peak calling of CBFβ-MYH11 fusion, RUNX1, and GATA2 ChIP-seq was conducted using MACS1.3.3^15^ at a *p*-value cutoff of 10^−6^. For DNA accessibility and H3K27ac data in inv(16) patients, the detailed analyses procedure was described as before^16^.

#### Super-enhancer identification

All H3K27ac peaks identified were filtered to exclude regions within ±2.0 kb around transcription start sites (TSSs), and then used as input for the ROSE algorithm^17^ to predict SEs.

#### Tag counting

Read counts for each putative region were enumerated and then normalized to RPKM (reads per kilobase of gene length per million reads) for visualization in heat maps or boxplots. For each base pair in the genome, the number of overlapping sequence reads was determined, averaged over a 10 bp window, and visualized in the UCSC genome browser (http://genome.ucsc.edu).

#### Motif analysis

Motif discovery was performed using GimmeMotifs^18^ with a threshold score of 0.9 (on a scale from 0 to 1).

#### Expression analysis

RNA-seq reads were uniquely mapped to the hg19 reference genome using STAR^19^, and subsequently normalized to RPKM values for all RefSeq genes using tag counting scripts. Gene-level count matrix was used as input for DESeq2 package^20^ to distinguish differentially expressed genes between any two groups. Significant genes were determined by a fold change cutoff of 1.5 and adjusted *p*-value of 0.01.

## Results

### CBFβ-MYH11 expressing cells harbor transcriptional characteristics of megakaryocytic and erythroid cells

**Fig. 1:**
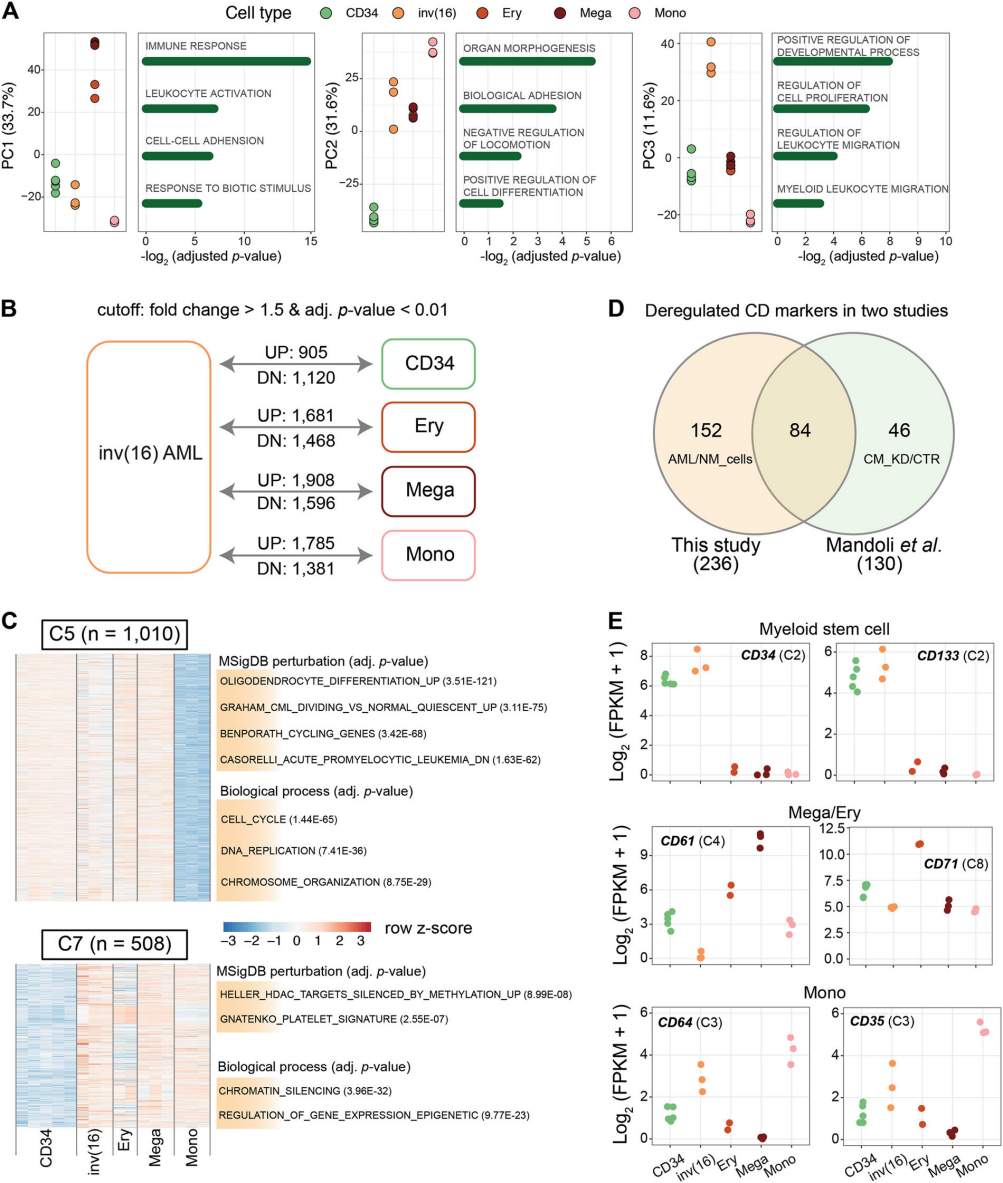
The deregulated gene expression programs of primary inv(16) AML blasts compared to four normal cell types. **a** The transcriptional relationship based on RNA-seq among cell types revealed by principal component analysis. The five cell types are CD34^+^ progenitor cell (CD34), primary inv(16) AML cell (inv(16)), erythrocyte (Ery), megakaryocyte (Mega), and monocyte (Mono). **b** Pairwise gene expression comparison between primary inv(16) cells with other cell types. **c** Two example clusters after defining distinct expression patterns by k-means clustering among five cell types (see also Supplementary Figure 1B). The raw *p*-value in functional enrichment is adjusted by the Benjamini–Hochberg procedure. **d** Overlap of differentially expressed cell markers between our previous study (Mandoli et al., 2014) and the present study. NM_cells: normal cell types (CD34, Mega, Ery, and Mono), CM_KD: CBFβ-MYH11-knockdown cells, CTR: control cells. **e** Transcriptional changes for several cell-type-specific markers. Labels in the parentheses indicate which cluster from Supplementary Figure 1B this gene is from

To examine the transcriptional differences between inv(16) AML patients expressing CBFβ-MYH11 and normal hematopoietic cell types, we used RNA-seq and compared global gene expression levels between AML blasts and normal CD34^+^ progenitor cells (CD34), megakaryocytes (Mega), erythrocytes (Ery), and monocytes (Mono). Unsupervised principal component analysis (PCA) revealed that inv(16) AMLs displayed different transcriptomic landscapes as compared to normal lineages (Supplementary Figure 1A). The main source of variability (PC1) was the difference between Mega/Ery and CD34/inv(16) cells (Fig. 1a), with the top contributing genes in PC1 mainly involved in immune response terms. In contrast, the second component (PC2) showed significant similarity between inv(16) and Mega/Ery, and was enriched in cell differentiation related terms (Fig. 1a). The transcriptional difference between inv(16) leukemic blasts and normal cells was revealed by PC3, which terms associated with leukocyte migration and activation. Each pairwise comparison between individual normal cell type and inv(16) blasts revealed more than 2000 differentially expressed genes (Fig. 1b, Supplementary Table 1). Together, these results suggest that inv(16) AMLs carry unique gene expression signatures (PC3), but also signatures that resemble CD34^+^ progenitor cells (PC1) or mature cells such as megakaryocytes (PC2).

Next, all differentially expressed genes were clustered into eight groups by the k-means clustering method (Supplementary Figure 1B, C). Besides cell-type-specific clusters (C1, C4, C6, and C8), we again found that inv(16) blasts shared gene signatures with normal cell types. For instance, the C2 cluster revealed similar expression patterns between inv(16) and CD34^+^ progenitors, while the inv(16) genes expressed in C3 resembled more closely a Mono signature. Furthermore, the primary inv(16) blasts shared a subset of gene signatures with Mega/Ery as shown in the C5 and C7 cluster (Fig. 1c), although these genes also displayed similar expression levels in CD34 or Mono. These findings indicate that inv(16) blasts maintained certain transcriptional signatures from progenitors, but also already obtained characteristics of mature cells.

Different hematopoietic cell types can be identified by examining expression of cluster of differentiation (CD) markers. Here, we identified 236 CD markers differentially expressed between inv(16) and normal cells. Our previous studies showed altered transcriptional activity of CD markers upon transient knockdown of CBFβ-MYH11 in ME-1 cells^11^. To examine whether the 236 differentially expressed CD markers from this study might be directly regulated by CBFβ-MYH11, we compared the two datasets. More than half of CBFβ-MYH11-dependent CD markers (84/130) were also differentially expressed in the comparison of normal cell types versus inv(16) AMLs (Fig. 1d), suggesting putative regulation by CBFβ-MYH11. Among these, myeloid stem cell markers like CD34 and CD133 displayed significantly higher expression levels in inv(16) AML than other mature cells (Fig. 1e). However, some markers of mature cells like CD64 and CD71 were also expressed in inv(16) AML, suggesting inv(16) cells might already be partly differentiated.

To extend this finding we examined several other marker genes involved in hematopoiesis including *GFI1B*, *RUNX1*, and *GATA2* (Supplementary Figure 2A). It has been shown that expression of *GFI1B* is key to megakaryocyte and platelet development^21^. Moreover, CBFβ-MYH11 binds a putative downstream regulatory element of *GFI1B* gene^11^ (Supplementary Figure 2B). We confirmed the highest transcriptional level of *GFI1B* in Ery/Mega, but also found that the inv(16) blasts expressed higher levels of *GFI1B* as compared to Mono, and similar to CD34 progenitors (Supplementary Figure 2A). Together, our results suggest that expression of CBFβ-MYH11 leads to impaired differentiation in part through deregulation of genes involved in maturation of megakaryocytic cells.

### Epigenomic clustering of inv(16) cells, megakaryocytes, and erythroblasts

**Fig. 2:**
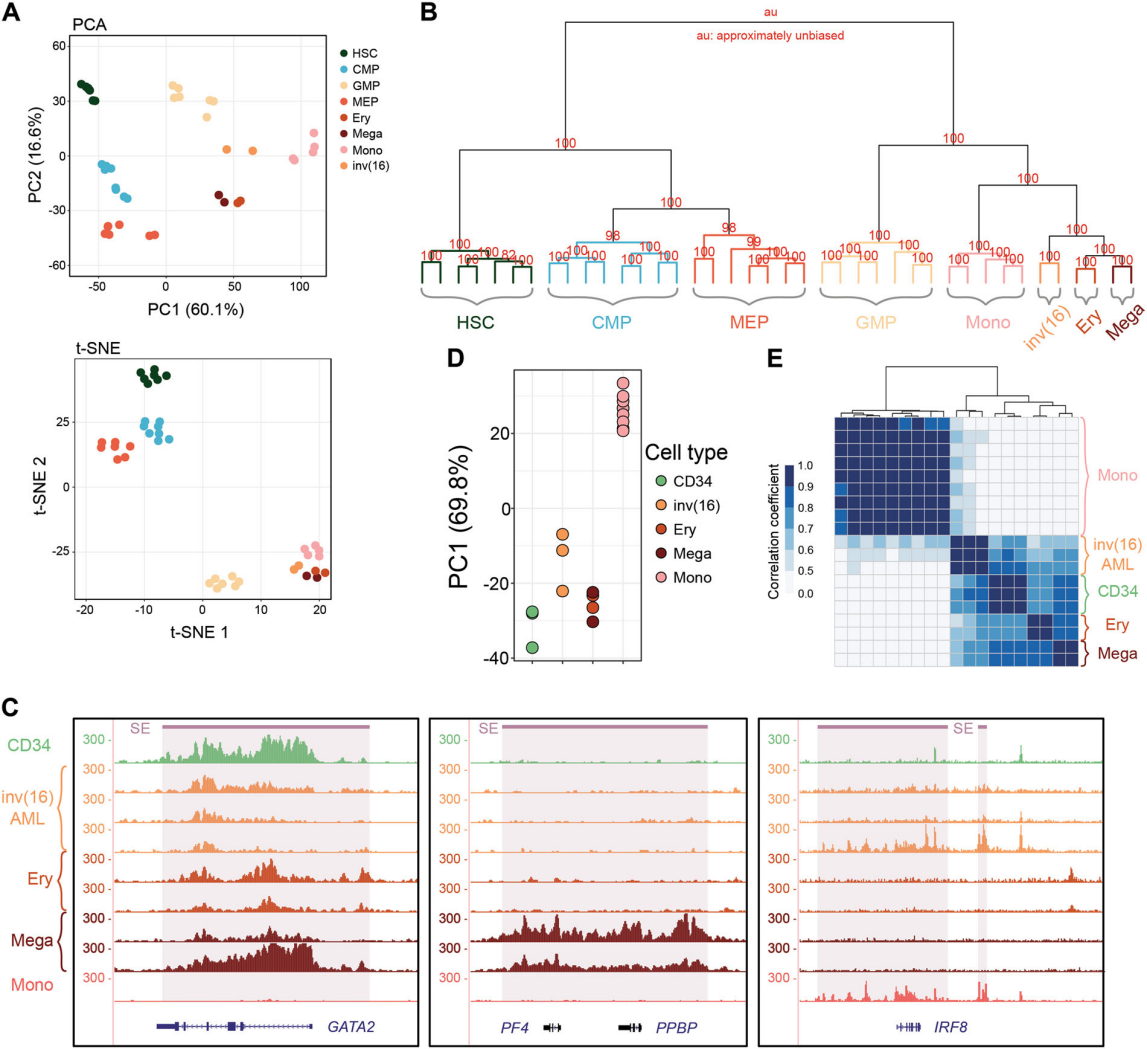
Primary inv(16) cells are epigenetically more similar to megakaryocytes and erythrocytes. **a** PCA and t-SNE analysis of DNA accessibility data display a clear separation along cell differentiation trajectories. Normal cell types include hematopoietic stem cell (HSC), common myeloid progenitor (CMP), granulocyte-macrophage progenitor cell (GMP), megakaryocyte-erythroid progenitor cell (MEP), erythrocyte (Ery), megakaryocyte (Mega), and monocyte (Mono). inv(16): primary inv(16) AML cell. **b** Hierarchical clustering of the top 5000 variable DNA accessibility sites. Numbers on the branch indicate bootstrap support scores over 1000 samplings. **c** Variable super-enhancer landscapes (H3K27ac ChIP-seq) in the *GATA2*, *PF4*/*PPBP* and *IRF8* loci. SE: super-enhancer. Average H3K27ac density of three CD34 cells and nine monocytes were calculated for better visualization. **d**, **e** Principal component 1 (PC1) and clustering plots of cell type relationship based on H3K27ac signal in super-enhancers

To further investigate whether cells blocked by CBFβ-MYH11 are poised for a certain lineage we compared epigenetic landscapes. For this, we generated DNaseI-seq and H3K27ac ChIP-seq profiles^16^ in inv(16) AML cells and compared those to profiles created in Mega, Ery, and Mono. In addition, we downloaded public ATAC-seq data^22^, which showed high consistency with DNaseI-seq (Supplementary Figure 3A), from several progenitor cell types including HSC, common myeloid progenitor, granulocyte–macrophage progenitor cell, and megakaryocyte-erythroid progenitor cell, to assess the similarity with inv(16) AMLs in open chromatin patterns. Principal component analysis (PCA), the *t*-distributed stochastic neighbor embedding (t-SNE) and hierarchical clustering results based on DNA accessibility showed robust classification of cell types and differentiation trajectory (Fig. 2a, b). Interestingly, the inv(16) AML cells displayed most similarity in open chromatin signatures and hence closer relationships with Mega and Ery cells (Fig. 2b, Supplementary Figure 3B).

Given that SEs are able to precisely capture cell identity^23^, we delved into genome-wide SEs landscapes using H3K27ac profiling. To integrate progenitor cells in our SE analysis, we downloaded H3K27ac data of CD34^+^ cells from NIH Roadmap Epigenomics^24^. The profiles of CD34, inv(16), Mega, Ery, and Mono demonstrated high-quality H3K27ac-binding patterns and cell-type-specific SEs profiles, for example at the *GATA2, PF4*, and *IRF8* loci (Fig. 2c, Supplementary Table 2). PCA analysis of H3K27ac signal at SEs confirmed these findings and showed that principal component 1 could clearly separate Mono from CD34, inv(16), Mega, and Ery, and also reveal relatively closer distance between inv(16) and Mega/Ery (Fig. 2d, Supplementary Figure 3C). Pearson clustering of SEs again uncovered preferential grouping of inv(16) cells with CD34, Mega, and Ery, while Mono SEs formed an individual cluster (Fig. 2e). Overall, these results suggest that consistent with our RNA-seq findings, inv(16) AML cells might carry a specific epigenetic state with high similarity to Mega/Ery cells, and represent cells that are putatively blocked along the Mega/Ery differentiation pathway.

### *CBFβ*-*MYH11* single oncogene expression blocks megakaryocyte/erythrocyte differentiation

**Fig. 3:**
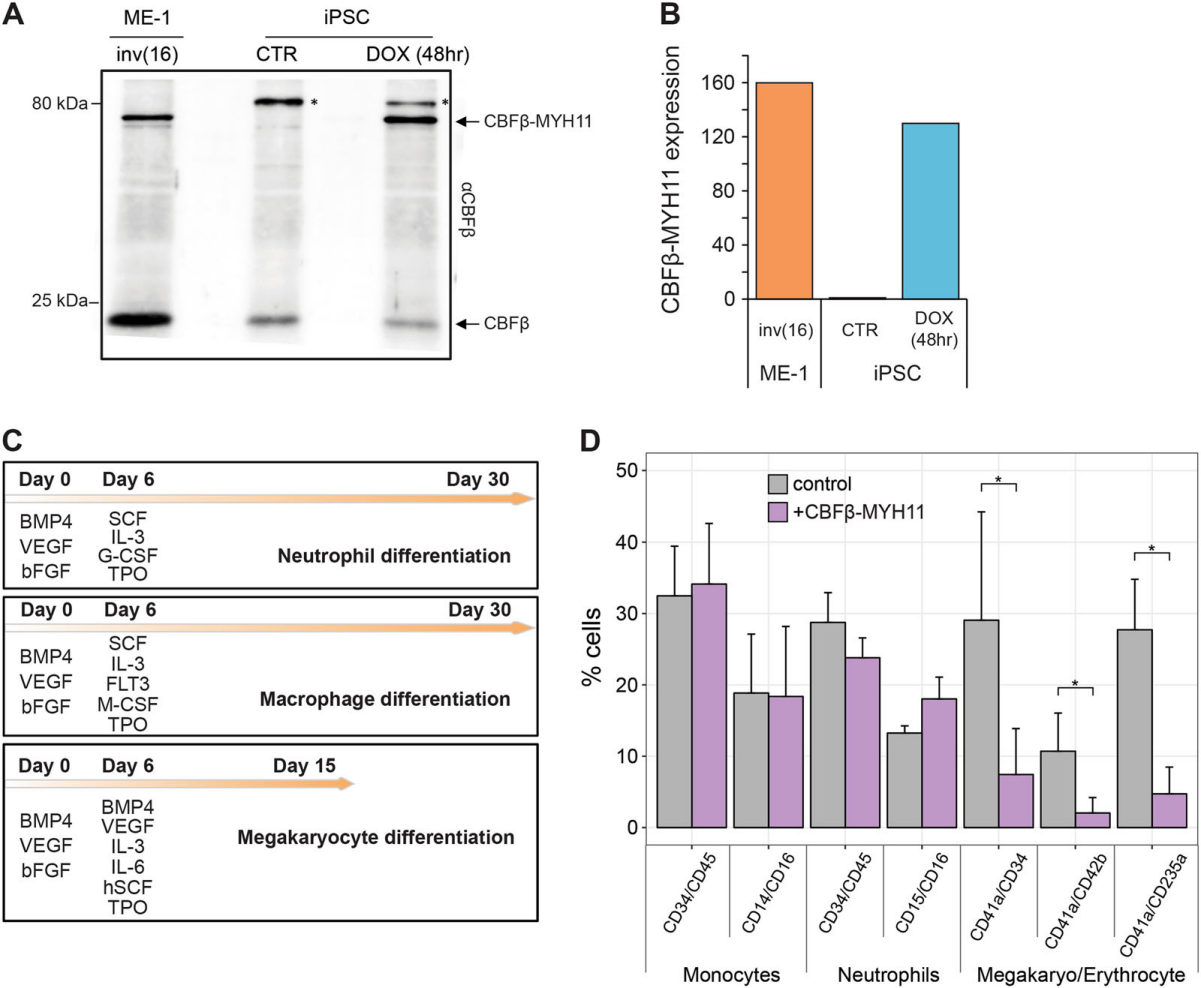
The *CBFβ*-*MYH11* oncogene blocks cell differentiation towards megakaryocyte/erythrocyte lineages. **a** Western blots of the CBFβ-MYH11 oncoprotein in the inv(16) AML ME-1 cell line and in the iPSC system before and after dox addition. Arrows point to the endogenous CBFβ and inducible expressed CBFβ-MYH11 protein. Asterisks represent aspecific binding of the antibody. Molecular weight markers in kilodaltons are listed on the left. **b** Expression levels of the *CBFβ-MYH11* oncogene in ME-1 cells and in iPSC without (CTR) or after dox induction of CBFβ-MYH11. **c** The methodology for iPSC differentiation towards neutrophil, macrophage, and megakaryocyte lineages. **d** The percentage of cells showing specific myeloid markers after differentiation following the protocols in **c**. The mean and standard deviation of cell fractions from four independent differentiation experiments are shown. A *p*-value of <0.05 was considered statistically significant by Wilcoxon signed-rank test: *p < 0.05

A major limitation when analyzing inv(16) cell lines and primary inv(16) AML blasts is that they harbor many additional mutations. To exclude the disturbance of other genetic lesions, we utilized an in vitro iPSC-based hematopoietic differentiation system in which the expression of a single oncogene can be induced with doxycycline (dox), as successfully conducted in our previous study^25^. The established system contains dox-inducible CBFβ-MYH11 (Fig. 3a, b) and we expressed the oncoprotein during differentiation towards the granulocytic, monocytic, and megakaryocyte lineage (Fig. 3c), allowing the investigation of the effects of CBFβ-MYH11 in the absence of additional leukemia driver mutations. Flow cytometry analysis of four independent experiments using iPS cells in which CBFβ-MYH11 expression was induced revealed a remarkable reduction in CD41a, CD42b, and CD235a positive cells during megakaryocyte/erythrocyte differentiation as compared to iPS cells not expressing CBFβ-MYH11 (Fig. 3d, Supplementary Figure 4). In contrast, no significant effects were observed during monocyte and granulocyte/neutrophil differentiation. Together, these results reflect that CBFβ-MYH11 has a dominant effect on blocking Mega/Ery differentiation, and suggest inv(16) cells might correspond to cells that are arrested in a progenitor stage of this lineage.

### CBFβ-MYH11 knockdown affects cell proliferation

**Fig. 4:**
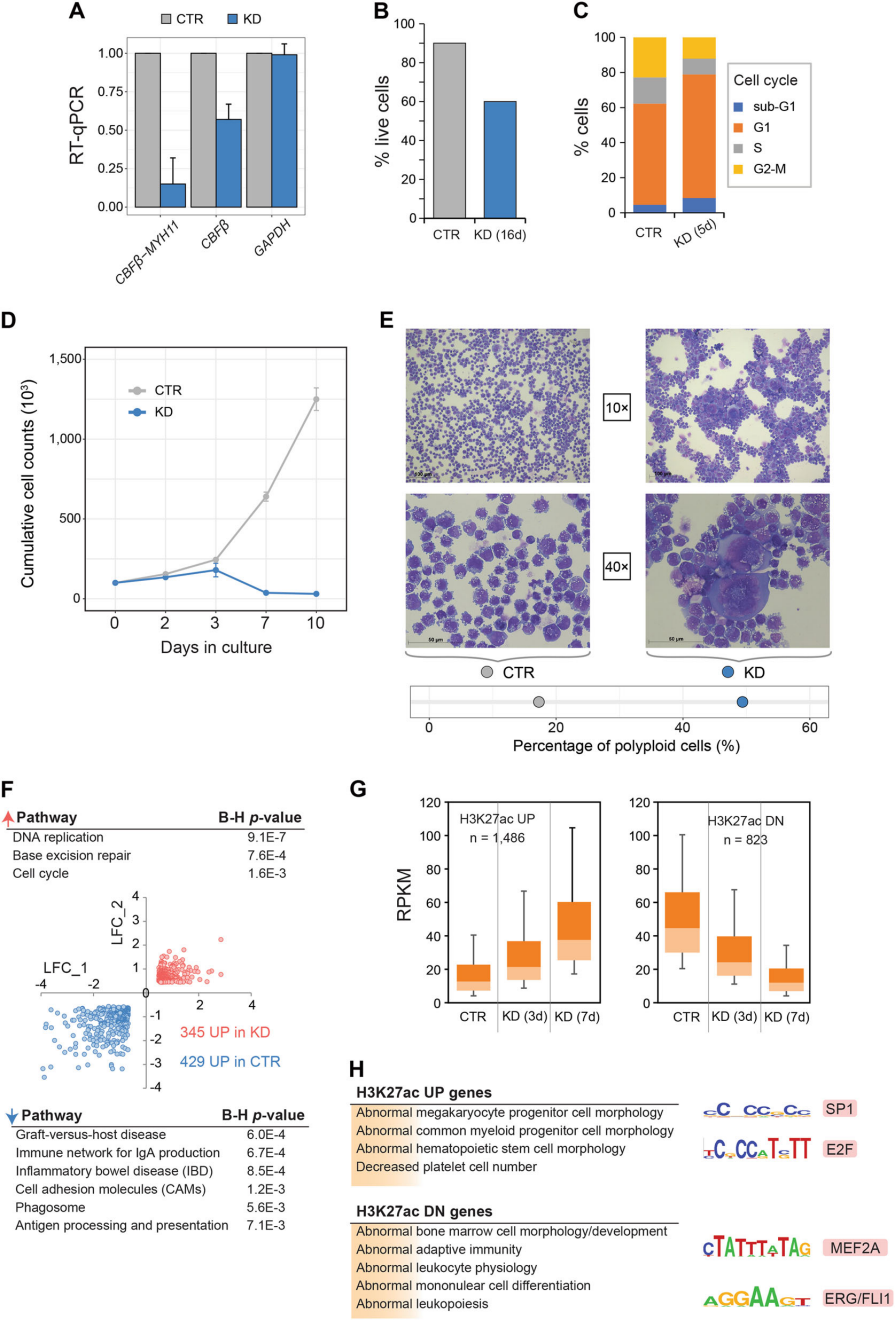
Cell cycle deregulation induced by CBFβ-MYH11 fusion. **a** RT-qPCR analysis of *CBFβ-MYH11* and *CBFβ* in ME-1 cells before and after CBFβ-MYH11 knockdown (KD). Data are normalized to *GAPDH* expression level. **b** Cell viability in response to CBFβ-MYH11 knockdown. **c** FACS analysis showing the percentage of cells in four cell cycle phases after CBFβ-MYH11 knockdown. **d** Cumulative cell counts in culture of ME-1 cells before and after CBFβ-MYH11 knockdown. **e** Cell morphology of ME-1 cells before and after CBFβ-MYH11 knockdown. In the KD group, we found more polyploid cells (49.4% compared to 17.3% in control) with increased size and decreased nuclear–cytoplasmic ratio. **f** Log_2_ fold change (LFC) of ME-1 CBFβ-MYH11 knockdown vs ME-1 control cells for a replicate RNA-seq experiment. Involved pathways of differentially expressed genes between control and CBFβ-MYH11-knockdown cells are indicated. **g** Differential H3K27ac enrichment before (CTR) and after (KD) CBFβ-MYH11 knockdown. **h** Functional annotation of genes and enriched motifs associated with differential H3K27ac peaks

To further explore the detailed functional pathways induced by CBFβ-MYH11 alone, we generated a stable dox-inducible CBFβ-MYH11-knockdown (KD) cell line based on the inv(16) cell line ME-1^26^) (Fig. 4a), and further examined genome-wide changes in the transcriptome as well as the acetylome. Previously, CBFβ-MYH11 transduction of CD34^+^ cells was shown to enhance proliferation^27^. In line with these results, knockdown of CBFβ-MYH11 in ME-1 cells affected proliferation, with more cells in the G1 phase and more cell adherence^11^, while a slight reduction in cell viability was observed (Fig. 4b, c). The latter finding was also supported by an increased number of cells in the sub-G1 cell phase, which is indicative of apoptotic or necrotic cell death (Fig. 4c, Supplementary Figure 5). To provide further evidence for the differentiation stage of the cells after CBFβ-MYH11 knockdown, we performed cell counting and morphology analysis. Delayed expansion for CBFβ-MYH11 KD cells was observed as compared to control after 3 days of culture (Fig. 4d). Furthermore, the morphology of KD cells by cytospin showed the presence of polyploid cells with increased size and decreased nuclear–cytoplasmic ratio that morphologically resemble megakaryocytes (Fig. 4e), again suggesting that these cells after CBFβ-MYH11 knockdown become more megakaryotic. Transcriptome analysis between control and CBFβ-MYH11-knockdown cells at day 5 corroborated the effect on cell cycle, with genes involved in controlling DNA replication and cell cycle pathways increased in expression level (Fig. 4f). Together these results suggest that CBFβ-MYH11 knockdown might trigger the onset of differentiation and cell cycle arrest^28^.

Given that altered transcriptional levels are a reflection of epigenetic changes, we performed H3K27ac ChIP-seq (positively correlated with gene expression) on control and CBFβ-MYH11 knockdown cells, and inspected differential H3K27ac peaks (>100 tags and two-fold difference). A total of 2309 differential regions were identified, around 16.2% of them were covered by SEs from inv(16) AML patients. The genes associated with regions showing increased acetylation after knockdown were functionally related to megakaryocyte differentiation and platelet formation (Fig. 4g, h), while genes assigned by loci going down in acetylation were involved in leukocyte physiology. Motif analysis in the two types of peaks revealed enrichment for MEF2A and ERG/FLI1 motifs in regions attenuated in H3K27ac signal (Fig. 4h, right). In contrast, H3K27ac-increased regions were significantly occupied by SP1 and E2F motifs, again suggesting deregulation of the DNA replication machinery^29^.

### CBFβ-MYH11 knockdown increases GATA2/KLF1 occupancy at RUNX1-binding sites

Given that we observed significant changes in transcription and H3K27ac occupancy after CBFβ-MYH11 knockdown, we set out to further excavate its regulatory mechanisms. As CBFβ-MYH11 binds the RUNX1 protein, it potentially interferes with the normal RUNX1-regulated gene program. To investigate the proteins which might be implicated in altering the transcriptional program, we performed DNA pull-down experiments using a specific nucleotide sequence bound by CBFβ-MYH11 that contains the RUNX1 core consensus motif TGTGGT (RUNX1 oligo), in control and CBFβ-MYH11-knockdown ME-1 cells (Fig. 4a). The principle is to use this DNA probe to pull-down not only direct interactors with this sequence, but also all associated proteins, which are then identified by mass spectrometry. When performing the pull-down experiment using cells in which CBFβ-MYH11 is expressed and the same cells in which CBFβ-MYH11 has been knocked down, you can, based on the ratio of protein binding, determine which proteins are binding to the RUNX1-motif oligo in a CBFβ-MYH11 dependent fashion.

**Fig. 5:**
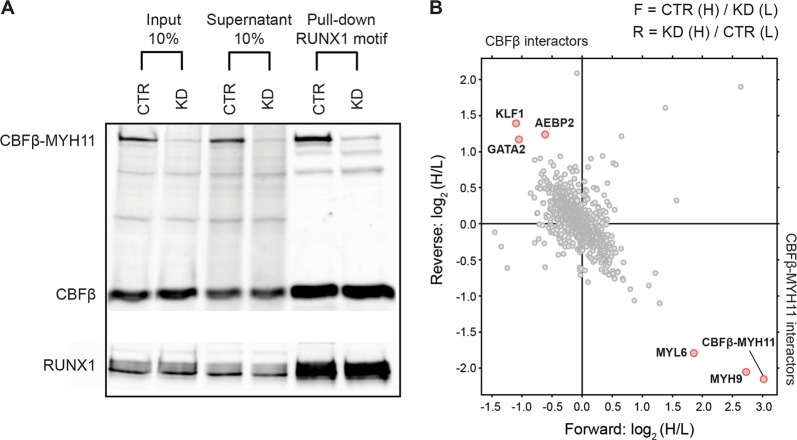
Identification of interactors affected by the CBFβ-MYH11 fusion. **a** Western blot analysis of a DNA pull-down experiment in ME-1 cells using CBFβ and RUNX1 antibodies. **b** Scatterplot showing the CBFβ-MYH11 interactome. Proteins interacting with a RUNX1 motif containing oligo before or after CBFβ-MYH11 knockdown are plotted by their SILAC-ratios in the forward (*x*-axis) and reverse (*y*-axis) SILAC experiment. Specific interactors with the RUNX1 motif containing oligo that bind upon CBFβ-MYH11 protein depletion lie close to the diagonal in the upper left quadrant. Interactors for which binding is CBFβ-MYH11 dependent are in the lower right quadrant

We could show that the oligonucleotide with the RUNX1 motif efficiently pulled down CBFβ-MYH11 as well as RUNX1 and CBFβ from an ME-1 cell lysate (Fig. 5a), whereas significantly attenuated affinity for CBFβ-MYH11 was observed when the knockdown cell lysate was used. Importantly, knockdown of CBFβ-MYH11 did not affect RUNX1 and wild-type CBFβ occupancy, suggesting changes in protein-binding profiles are likely due to the absence of CBFβ-MYH11.

To decipher the interactome of the CBFβ-MYH11 complex at RUNX1-binding sites, we employed previously described SILAC-based technology^11,30^, using extracts derived from CBFβ-MYH11-knockdown ME-1 cells grown in light (L) or heavy (H)-labeled medium, incubated with oligonucleotides containing the RUNX1 motif (see Methods section). Of the >1300 identified proteins at a high confidence level, only a limited number displayed highly significant SILAC-ratios (>2) as CBFβ-MYH11 interactors. The CBFβ-MYH11 complex seems to facilitate the recruitment of MYH9 and MYL6 proteins to RUNX1 sites, as shown by their enrichment in the pull-down assay from ME-1 lysates (Fig. 5b). Interestingly, upon CBFβ-MYH11 knockdown, two transcription factors (TFs), GATA2 and KLF1, are strongly enriched at RUNX1 occupancy loci, implying that these might play a crucial role in coordinating the RUNX1-dependent regulatory network in normal cells. Our previous findings demonstrated that *GATA2* and *KLF1* displayed enhanced levels in transcription after CBFβ-MYH11 knockdown^11^, and the present work could confirm not only increased RNA expression, but also stronger binding to the RUNX1 containing oligo by western blot (Supplementary Figure 6A, B), suggesting that the two TFs are repressed and might be replaced by CBFβ-MYH11 fusion in inv(16) AML. These findings are further supported by our previous inability to detect GATA2 and KLF1 as binders of RUNX1 sites^11^, as these assays were also done in the presence of CBFβ-MYH11.

### GATA2/CBFβ-MYH11 switching might drive megakaryocyte/erythrocyte differentiation

**Fig. 6:**
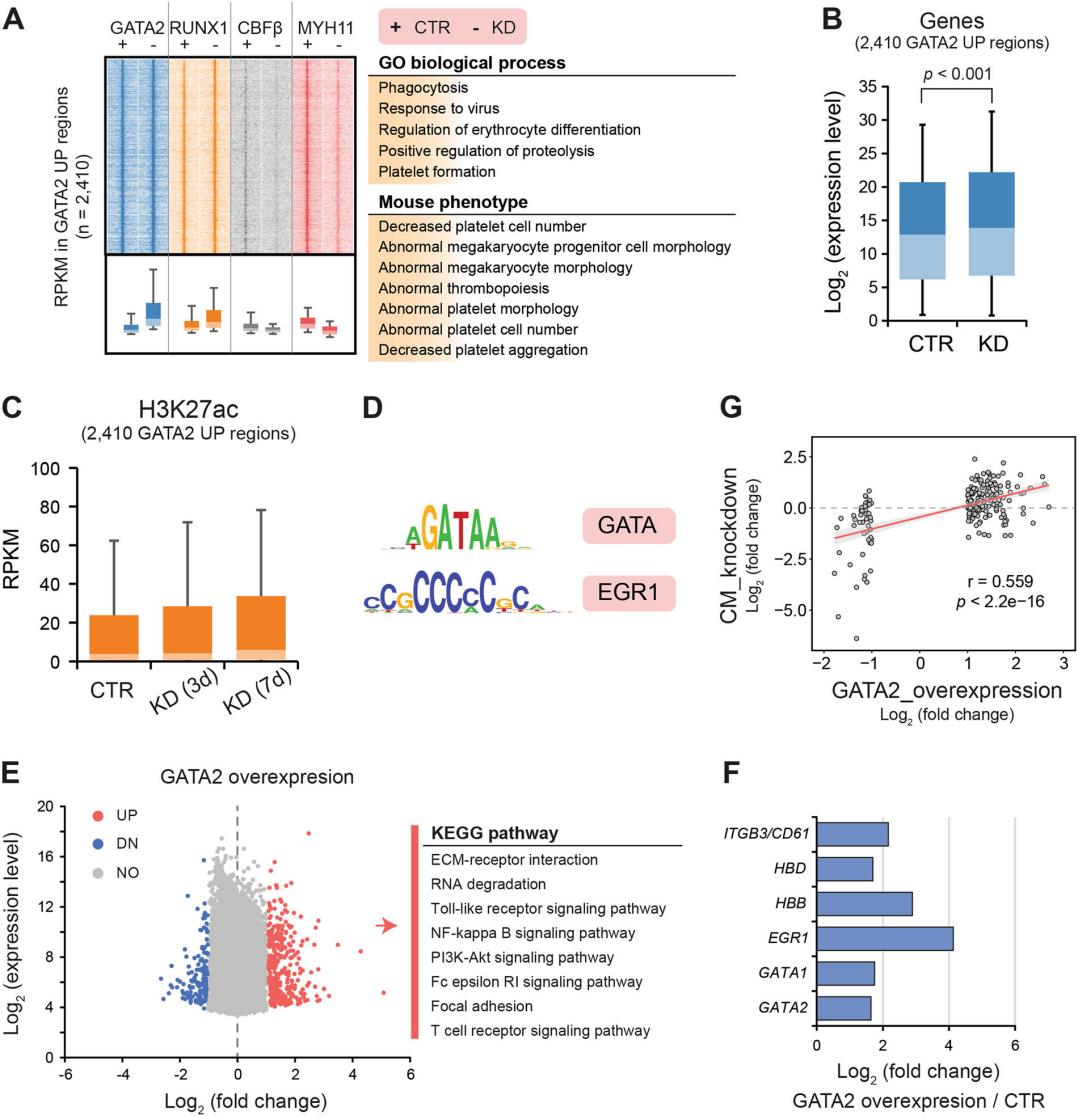
Overexpression of GATA2 can partly induce a gene program involved in megakaryocyte-directed differentiation. **a** Associated regulators and functional enrichment (ChIP-seq) in regions with increased GATA2 intensity after CBFβ-MYH11 knockdown (KD). **b**, **c** Genomic loci with elevated GATA2 signal show higher expression levels (**b**) of associated genes and H3K27ac occupancy (**c**) in the CBFβ-MYH11-knockdown cells. **d** Motif enrichment in regions with increased GATA2 density. **e** Gene changes upon GATA2 expression in ME-1 cells. (right) Enriched KEGG pathways of differentially expressed genes after enforced GATA2 expression. **f** Upregulated cell marker genes induced by GATA2 overexpression. **g** Correlation of deregulated genes between ME-1 cells overexpressing GATA2 and ME-1 cells upon CBFβ-MYH11 knockdown. Pearson correlation coefficient and *p*-value are shown

As GATA2 is the highest expressed GATA factor in ME-1 cells, we investigated the genome-wide binding pattern by ChIP-seq in control and CBFβ-MYH11-knockdown inv(16) cells, and also examined the RUNX1-binding profile in parallel. The genome-wide screen indicated that GATA2 occupied similar genomic regions as RUNX1 at most locations. Quantifying GATA2 occupancy at RUNX1 loci revealed 2410 sites with increased GATA2 signal and only 25 reduced binding sites. The 2410 regions showed increased RUNX1 occupancy but reduced CBFβ-MYH11 binding (Fig. 6a), and associated genes were involved in megakaryocyte/erythrocyte differentiation and platelet formation. Furthermore, genes associated with the 2410 regions were increased in expression levels, which was further reflected by elevated occupancy level of the H3K27ac mark (Fig. 6b, c), indicating activation of the GATA2-target gene program after the knockdown of CBFβ-MYH11. As could be expected, motif discovery at these regions presented remarkable enrichment for the GATA motif (Fig. 6d). In addition, also overrepresentation for the EGR motif was detected, suggesting that TFs binding this motif are potentially involved in modulating the gene transcription process in a synergistic manner (Fig. 6d).

To probe whether enforced GATA2 expression is sufficient to switch on this gene program, we transduced ME-1 cells with a GATA2 expression construct and performed global RNA-seq analysis. During hematopoiesis, overexpression of GATA2 shows a decrease in cell proliferation and induces differentiation toward the megakaryocyte lineage^31^. Here, global transcriptional analysis identified a total of 367 genes increased and 171 genes decreased in transcriptional levels after GATA2 overexpression (Fig. 6e). As expected, these upregulated genes contained GATA2 and several specific marker genes linked to megakaryocytic/erythrocyte differentiation, such as *CD61*, *HBD*, *HBB*, *EGR1*, and *GATA1* (Fig. 6f), and were mainly associated with various signaling pathways. Comparing transcriptomic changes after GATA2 overexpression with those observed after CBFβ-MYH11 knockdown displayed moderate correlation (*r* = 0.559) based on relative expression ratio, and only showed the overlap of 20 genes (5.4%) increased in expression and 19 genes (11.1%) decreased. These results suggest that enforced GATA2 expression alone can partly reboot the gene program involved in megakaryocyte-directed differentiation, despite insufficiency to completely mimic CBFβ-MYH11 knockdown.

Together, these findings suggest that activation of the GATA2-involved regulatory program after CBFβ-MYH11 knockdown is central but not sufficient in driving further differentiation, which is potentially orchestrated in collaboration with other factors like KLF1 and/or EGR1.

## Discussion

The *CBFβ-MYH11* fusion arising from inv(16) rearrangement was reported to lead to differentiation blockage of normal myeloid cells and result in AML, but whether this fusion blocks specific cell commitment has not been discerned. Here, we compared the global landscapes of gene expression, DNA accessibility, and H3K27ac between primary inv(16) AML blasts and normal cell types, and further investigated the functional contributions of CBFβ-MYH11 and its involved regulatory program in vitro.

Comprehensive transcriptomic exploration in vivo not only consolidated our previous findings^11,12,32^, but pinpointed towards unique gene expression programs underlying inv(16) AML cells. Further epigenomic analyses indicated more shared epigenetic determiners between inv(16) AML blasts and Mega/Ery cells, suggesting that primary inv(16) AML cells might be epigenetically primed for Mega/Ery differentiation with some key transcriptional pathways blocked. Therefore, in inv(16) leukemogenesis, the CBFβ-MYH11 fusion might skew leukemic blasts towards the Mega/Ery phenotype by epigenetic predisposition^33^ or be involved in setting up a specific Mega/Ery differentiation block by altering the cells gene program.

We have shown previously that CBFβ-MYH11 could activate transcription of self-renewal genes, and also repress expression of differentiation markers in the context of the cell line model ME-1^11^. Here, we used in vitro models with a single mutation (expression of CBFβ-MYH11) for surveying the role of CBFβ-MYH11. Using an iPSC system with dox-inducible CBFβ-MYH11 revealed that this fusion is able to specifically block in vitro Mega/Ery differentiation, but hardly has any effects on granulocyte and monocyte maturation. This finding suggests expression of CBFβ-MYH11 affects specific cell differentiation pathways.

Previous studies have proposed that CBFβ-MYH11 impairs normal binding of other proteins due to the higher affinity with RUNX1, and alters expression of RUNX1-dependent target gene sets^11,12,34^. We found that *GATA2* and *KLF1* showed elevated expression levels^11^ and also acted as significant interactors of RUNX1 after CBFβ-MYH11 knockdown, suggesting that CBFβ-MYH11 alters normal transcriptional programs of the two regulators and competitively takes over their binding sites at RUNX1 occupancy loci. GATA2 and KLF1 exert key effects in the Mega/Ery lineage fate decision via interaction with other modulators^35–37^. Our RNA-seq analysis revealed attenuated expression levels of *GATA2* and *KLF1* in inv(16) blasts as compared to Mega/Ery, and that *GATA2* overexpression could partly reboot a megakaryocyte-like gene program. As such, in line with previous reports^38^, weak expression of *GATA2* might be essential for inv(16) leukemia, and responsible for partial myelomonocytic differentiation featured by occasional cells of “hybrid” nature (nuclear characteristics of monocytes and both basophilic and eosinophilic granules), but increased GATA2 levels are needed for further differentiation. Focusing on GATA2, ChIP-seq illuminated its transcriptional activation role at these RUNX1-dependent target genes by increased recruitment of histone acetyltransferase activity and putative other TFs like EGR1 (refs. ^39–41^). Consequently, the data suggest that the GATA2-involved binding/regulatory program might be obstructed by the CBFβ-MYH11 fusion, leading to block of differentiation towards Mega/Ery in inv(16) leukemogenesis.

Megakaryocyte and erythrocyte share plenty of common molecular signatures, but the two cell types also exhibit distinct patterns in subtle regulatory networks^35,42^. High transcription of *GATA2* has been reported to solely promote megakaryocyte differentiation and suppress erythroid maturation^43^. In our study, GATA2 overexpression boosted expression levels of some typical marker genes driving Mega differentiation but did not display a strong correlation with results from CBFβ-MYH11 knockdown. This finding reveals that CBFβ-MYH11 interferes with coordinated orchestration of a precise balance of multiple factors in maturing Mega/Ery, but the enforced expression of GATA2 only recapitulates megakaryocyte differentiation during bifurcation of the two lineages. As a consequence, GATA2 alone is not sufficient to inhibit CBFβ-MYH11-caused leukemia, and it maybe has greater functional relevance only in context with overexpression of other regulators like KLF1.

In summary, our study suggests that the CBFβ-MYH11 fusion maintains inv(16) AML cells by attenuating expression levels of GATA2 and blocks their further differentiation towards Mega/Ery lineages via interfering with a GATA2/KLF1-involved regulatory network. Collectively, these results corroborate our previous findings, facilitate a better molecular understanding of the role of CBFβ-MYH11 in the pathogenesis of leukemia, and might ultimately help to improve therapy decision of inv(16) AML by designing specific (epi)drugs to reprogram *GATA2* or other target genes.

## Supplementary information

Supplementary Figure 1

Supplementary Figure 2

Supplementary Figure 3

Supplementary Figure 4

Supplementary Figure 5

Supplementary Figure 6

Supplementary Information

Supplementary Table 1

Supplementary Table 2
