# Supplementary figures and images for: CBFβ-MYH11 interferes with megakaryocyte differentiation via modulating a gene program that includes GATA2 and KLF1
Source: Blood Cancer J. 2019 Mar 8;9(3):33. doi: 10.1038/s41408-019-0194-8 (PMC6408575; doi:10.1038/s41408-019-0194-8)

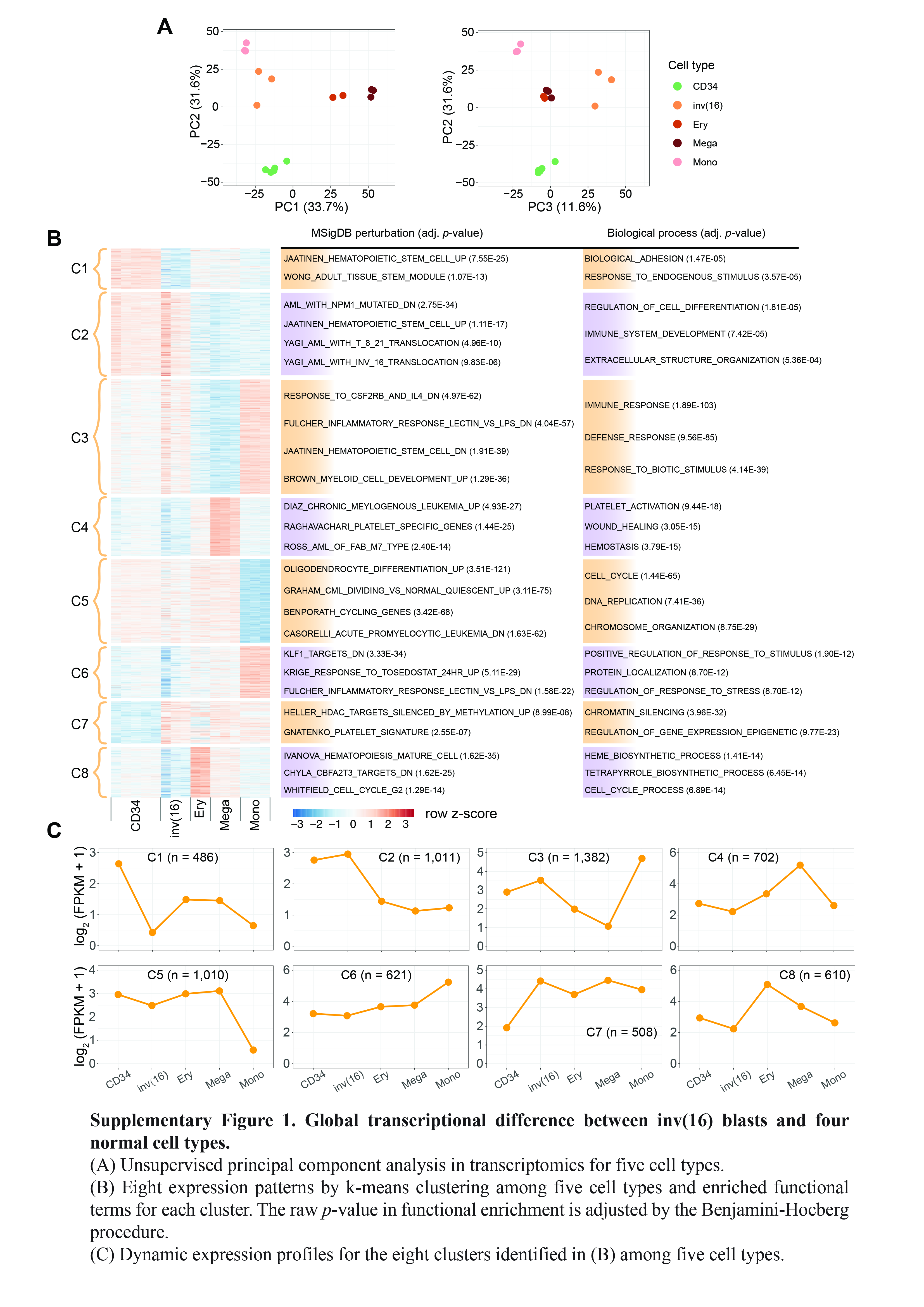

Supplement: Supplementary file 1 — Supplementary Figure 1 [file 41408_2019_194_MOESM1_ESM.tif]

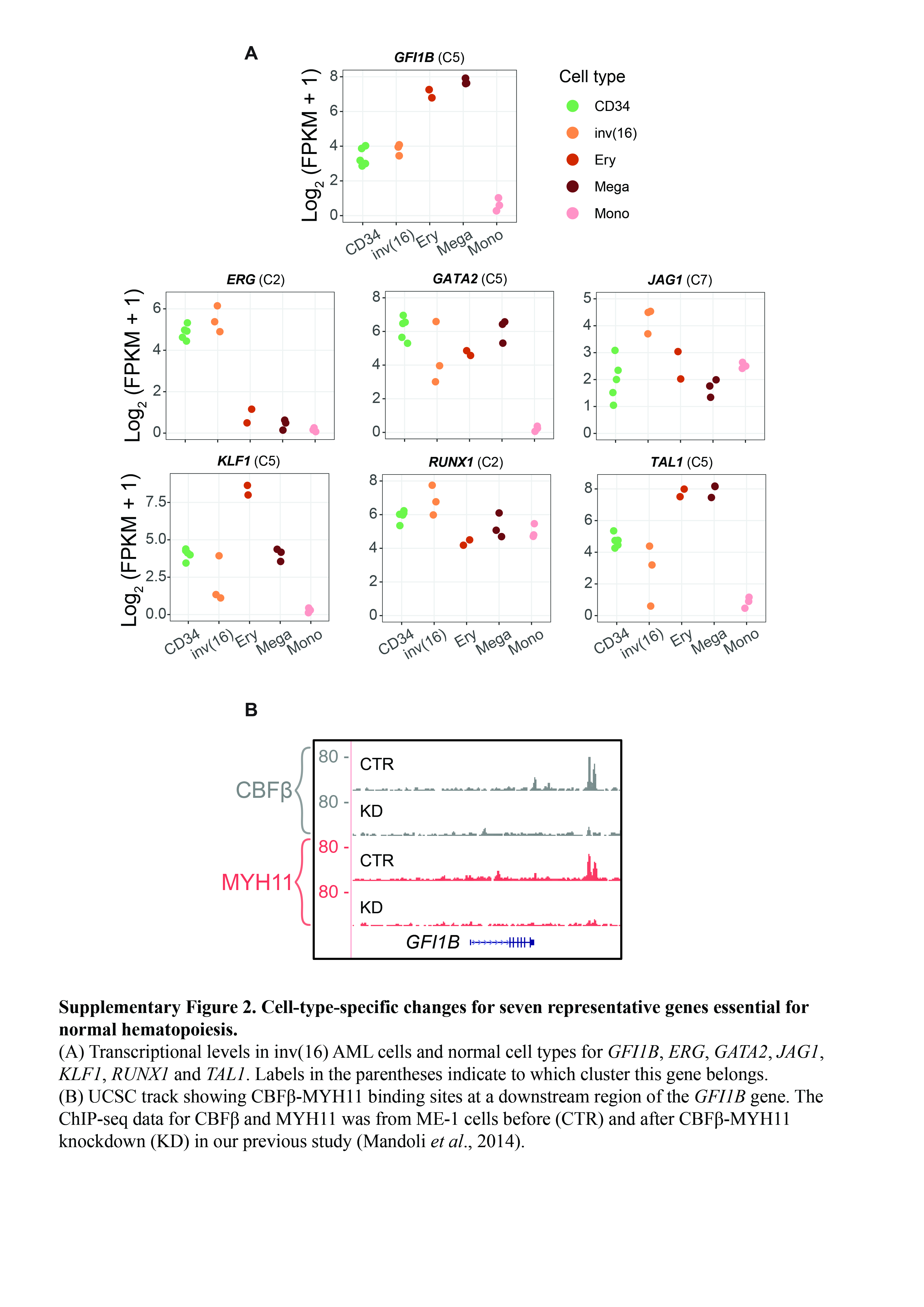

Supplement: Supplementary file 2 — Supplementary Figure 2 [file 41408_2019_194_MOESM2_ESM.tif]

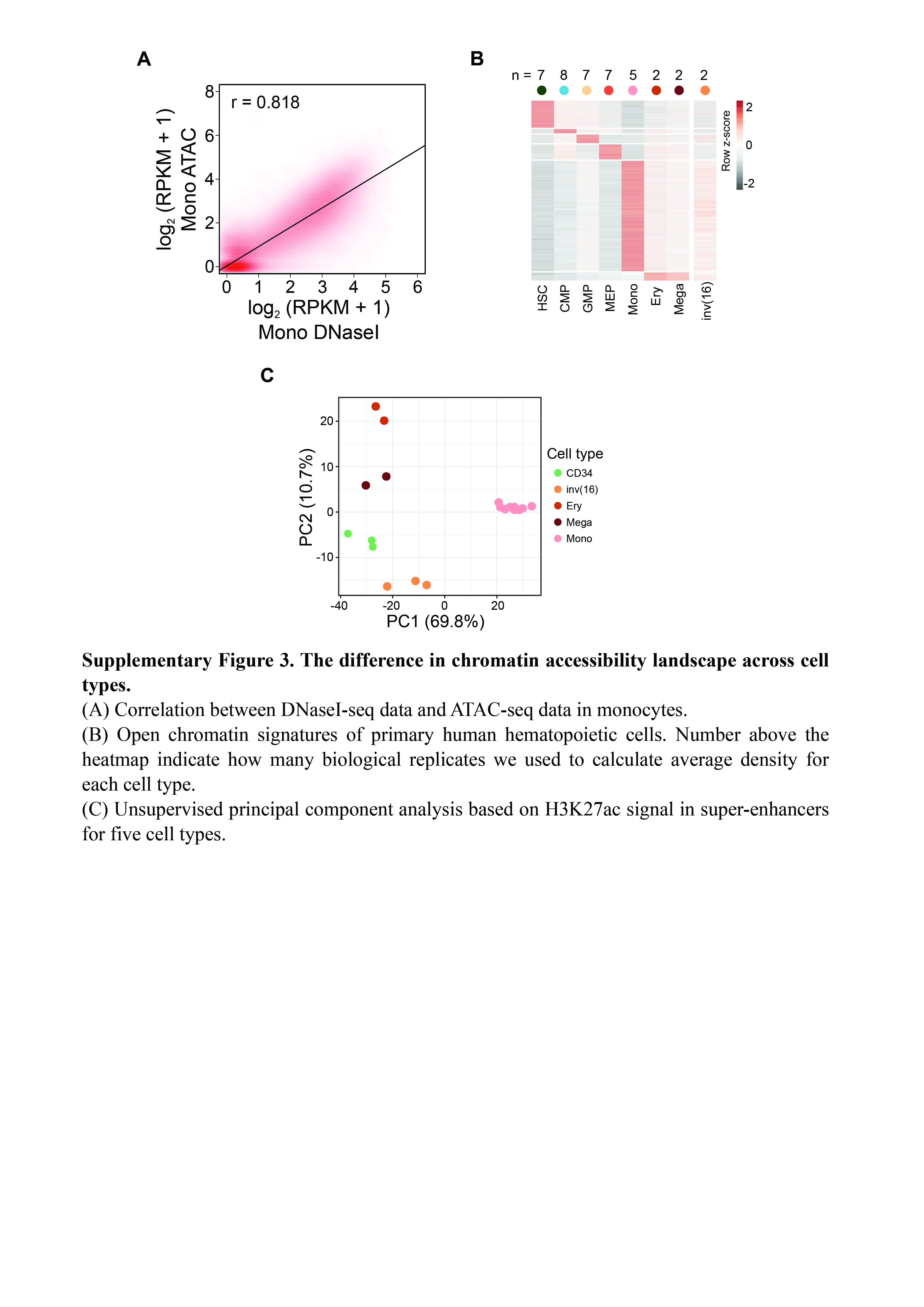

Supplement: Supplementary file 3 — Supplementary Figure 3 [file 41408_2019_194_MOESM3_ESM.tif]

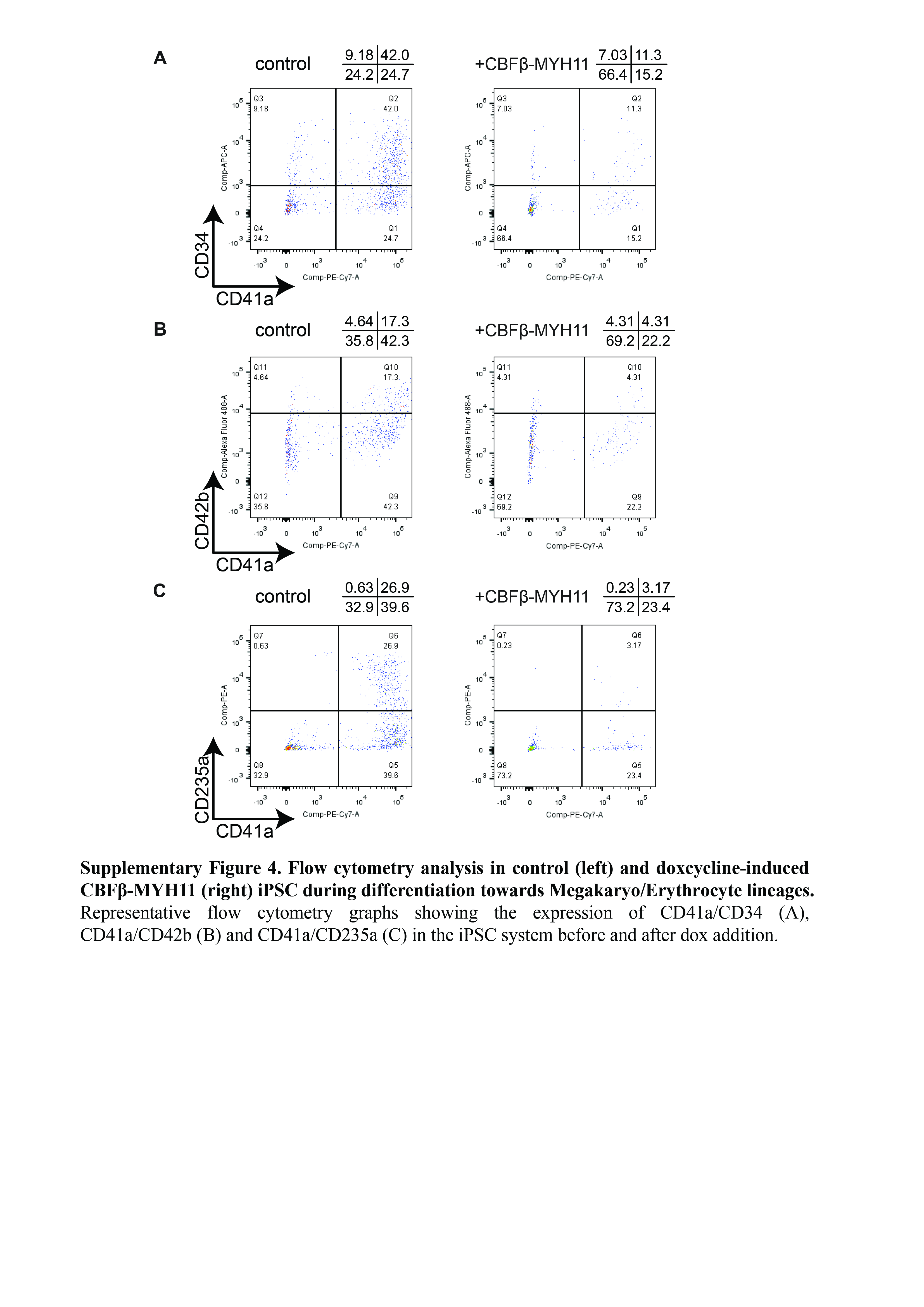

Supplement: Supplementary file 4 — Supplementary Figure 4 [file 41408_2019_194_MOESM4_ESM.tif]

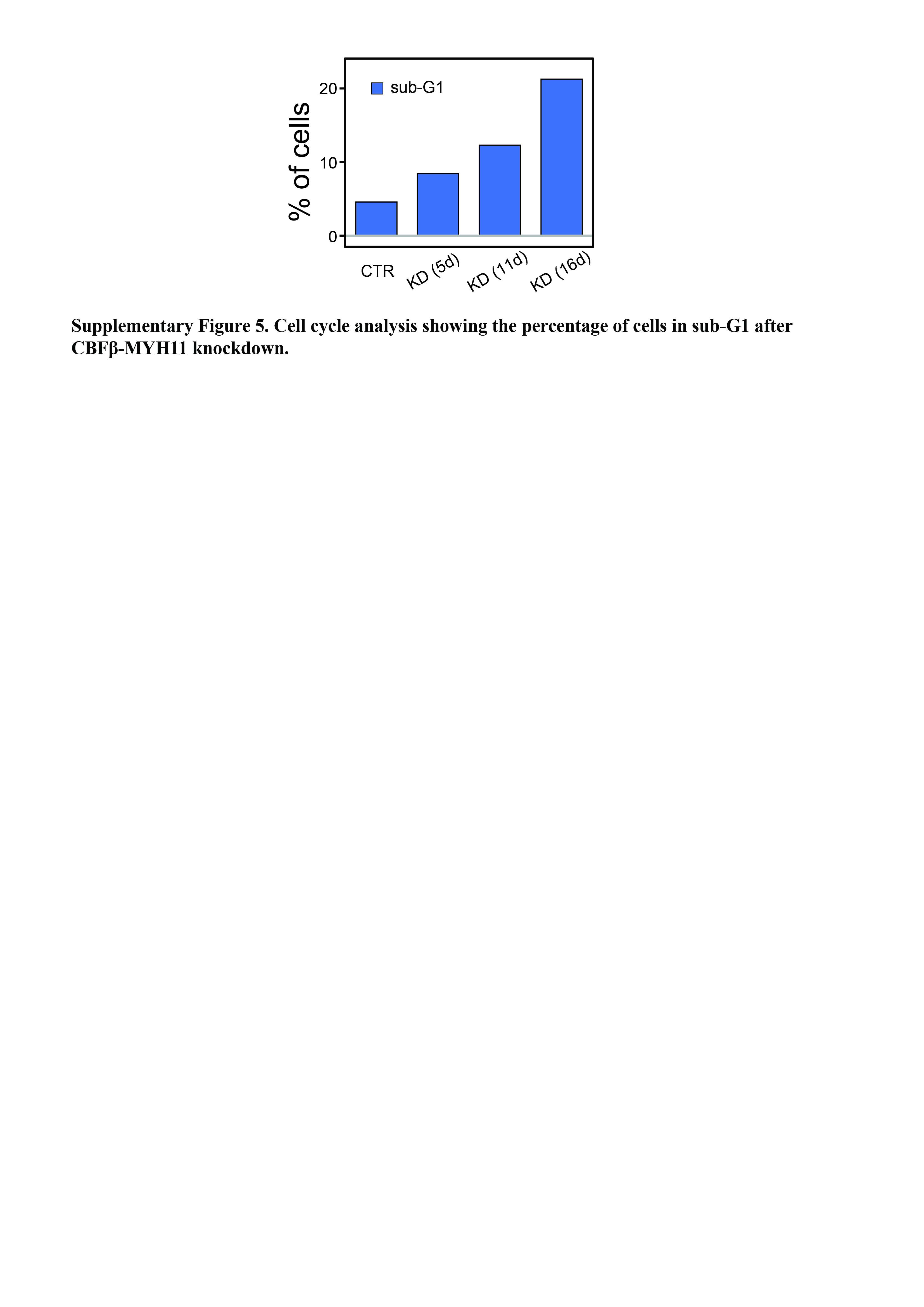

Supplement: Supplementary file 5 — Supplementary Figure 5 [file 41408_2019_194_MOESM5_ESM.tif]

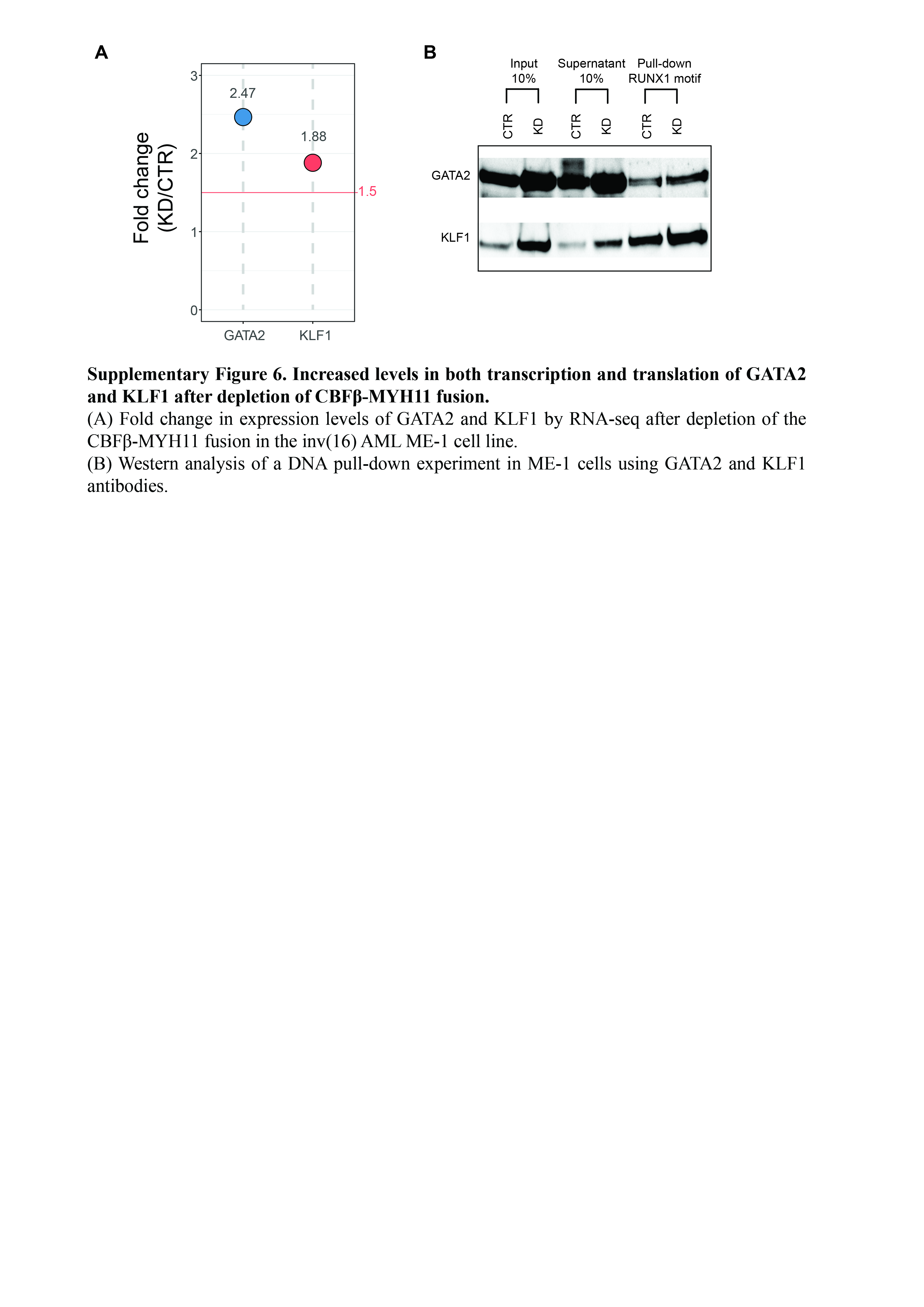

Supplement: Supplementary file 6 — Supplementary Figure 6 [file 41408_2019_194_MOESM6_ESM.tif]
