# Supplementary material for: CBFβ-MYH11 interferes with megakaryocyte differentiation via modulating a gene program that includes GATA2 and KLF1
Source: Blood Cancer J. 2019 Mar 8;9(3):33. doi: 10.1038/s41408-019-0194-8 (PMC6408575; doi:10.1038/s41408-019-0194-8)
Supplement: Supplementary file 7 — Supplementary Information [file 41408_2019_194_MOESM7_ESM.docx]

**Supplementary information**

**Characteristics of inv(16) patients**

Mononuclear CD34^+^ inv(16) AML blasts were isolated from bone marrow or peripheral blood of three *de novo* AML patients for subsequent processing. The study and sample usage were approved by the ethics committees of the contributing institutions.

**Isolation of erythrocyte, megakaryocyte and monocyte**

A whole unit (460 ml) of peripheral blood or a large unit ($>$ 120 grams) of cord blood were used. Diluted blood was separated by gradient centrifugation (Percoll 1.078 g/ml) and neutrophils were isolated from the pellet, after red blood cell lysis, by CD16 positive selection (Miltenyi). Leukocytes were further fractionated to obtain a monocyte rich layer using a second gradient (Percoll 1.066 g/ml). Monocytes were purified using CD16 depletion and subsequent a positive selection for the CD14-positive monocytes (Miltenyi). Whereas, megakaryocytes and erythroblasts were obtained by *in vitro* culture of cord blood HSC, and purified by CD34^-^/Lair1^-^/CD41^+^/CD42^+^/GPVI^+^ and CD36^+^/CD71^+^/CD235a^+^ markers separately. The purity of each preparation was assessed by flow cytometry on a Beckman Coulter FC500, by expression with Illumina HT-12v4 arrays (E-MTAB-1573 at arrayexpress) and finally by microscopic inspection of morphology using appropriate stained cytospin preparations. Samples which did not meet predefined criteria of cell purity were not included in the analysis. The purified cells were processed for subsequent multi-omics sequencing. Detailed versions of all protocols are available on the BLUEPRINT website (http://www.blueprint-epigenome.eu/).

**ChIP-seq**

Purified cells were first cross-linked with 1% formaldehyde for 20 min at room temperature, quenched with 0.125 M glycine and washed with three buffers: (i) PBS, (ii) buffer of composition 0.25% Triton X-100, 10 mM EDTA, 0.5 mM EGTA, 20 mM HEPES pH 7.6 and (iii) 0.15 M NaCl, 10 mM EDTA, 0.5 mM EGTA, 20 mM HEPES pH 7.6. Cells were then suspended in ChIP incubation buffer (0.15% SDS, 1% Triton X-100, 150 mM NaCl, 10 mM EDTA, 0.5 mM EGTA, 20 mM HEPES pH 7.6) and sonicated using a Diagenode Bioruptor sonicator for 20 min at high power (30 seconds ON and 30 seconds OFF cycle). Sheared chromatin was centrifuged at maximum speed for 10 min and then incubated overnight at 4$℃$ in incubation buffer supplemented with 0.1% BSA with protein A/G-Sepharose beads (Santa Cruz) and 1 µg of antibody. Beads were washed sequentially with four different wash buffers at 4$℃$: two times with a solution of composition 0.1% SDS, 0.1% DOC, 1% Triton, 150 mM NaCl, TEE (10 mM Tris pH 8.0, 0.1 mM EDTA and 0.5 mM EGTA), one time with a similar buffer but now with 500 mM NaCl, one time with a solution of composition 0.25 M LiCl, 0.5% DOC, 0.5% NP-40, TEE and two times with TEE. Precipitated chromatin was eluted from the beads with 400 µl of elution buffer (1% SDS, 0.1 M NaHCO_3_) at room temperature for 20 minutes. Protein-DNA crosslinks were reversed at 65$℃$ for 4 hours in the presence of 200 mM NaCl, after which DNA was isolated by Qiagen column. Chromatin isolation and ChIP-seq from primary inv(16) AMLs, monocytes, erythrocytes, and megakaryocytes was done according to Blueprint protocols ([www.blueprint-epigenome.eu/)](http://www.blueprint-epigenome.eu/)). Antibodies and primers for qPCR can be found below, and relative occupancy was calculated as fold change over the background, for which the second exon of the Myoglobin gene or the promoter of the *H2B* gene was used.

**RNA-seq**

Total RNA was isolated with the RNeasy RNA extraction kit with on-column DNaseI treatment (Qiagen), and the concentration was measured with a Qubit fluorometer (Invitrogen). Ribosomal RNA was removed by Ribo-Zero rRNA Removal Kit (Epicentre) according to manufacturer instructions. 16 µl of purified RNA was fragmented by addition of 4 µl 5$\times$ fragmentation buffer (200 mM Tris-acetate pH 8.2, 500 mM potassium acetate and 150 mM magnesium acetate) and incubated at 94$℃$ for exactly 90 s. After ethanol precipitation, fragmented RNA was mixed with 5 µg random hexamers, followed by incubation at 70$℃$ for 10 min and chilling on ice. We synthesized the first-strand cDNA with this RNA primer mix by adding 4 µl 5$\times$ first-strand buffer, 2 µl 100 mM DTT, 1 µl 10 mM dNTPs, 132 ng of actinomycin D, 200 U SuperScript III, followed by 2 h incubation at 48$℃$. First strand cDNA was purified by Qiagen mini elute column to remove dNTPs and eluted in 34 µl elution buffer. Second-strand cDNA was synthesized by adding 91.8 µl, 5 µg random hexamers, 4 µl of 5$\times$ first-strand buffer, 2 µl of 100 mM DTT, 4 µl of 10 mM dNTPs with dTTP replaced by dUTP, 30 µl of 5$\times$ second-strand buffer, 40 U of Escherichia coli DNA polymer­ase, 10 U of E. coli DNA ligase and 2 U of E. coli RNase H, and incubated at 16$℃$ for 2 h followed by incubation with 10 U T4 polymerase at 16$℃$ for 10 minutes. Double-stranded cDNA was purified by Qiagen mini elute column and used for library preparation as described in the KAPA HyperPrep protocol. We incubated 1 U USER (NEB) with adaptor-ligated cDNA at 37$℃$ for 15 min followed by 5 min at 95$℃$ before PCR.

**Illumina high-throughput sequencing**

ChIP-seq and RNA-seq libraries were loaded on E-gel and a band corresponding to ~300 bp (DNA $+$ Adaptor) was collected. After quality assessment, the eligible library was sequenced on the Illumina HiSeq 2000 machine and generated 42 to 52 bp tags. All ChIP-seq and RNA-seq data can be downloaded from the Gene Expression Omnibus (GSE117138), or the Blueprint DCC (<http://dcc.blueprint-epigenome.eu/#/files)>

**Cell culture**

ME-1 cells were routinely cultured in RPMI 1640 supplemented with 10% FCS and 1% pen/strep at 37$℃$. ME-1 shRNA stable cell lines were cultured in tet-free FBS, and shRNA expression was induced for 72 h for RT qPCR and protein lysate preparation and 7 days for cell cycle analysis by adding 600 ng/ml doxycycline (Dox). Induced pluripotent stem cells (iPSCs) were generated from megakaryoblast based on previous protocol^1^ at the Sanquin Research Department of Hematopoiesis, Amsterdam, The Netherlands. CBF$\beta$-MYH11-expressing iPSCs were generated by using a previously described strategy of knock-in using an AAVS1 homology donor vector and CRISPR-Cas9^2, 3^. Briefly, two million iPSCs were nucleofected with donor vector containing an inducible promoter for expression of the cloned gene^4^ and a gene targeting vector for the AAVS1 locus^2^. Transfected cells were plated in one well of a vitronectin (Life Technologies) coated six-well plate in E8 media (Life technologies) supplemented with 10 µM of rock inhibitor for 24 h. Cells were dissociated using accutase (Life Technologies) and seeded at low density on a vitronectin coated dish in E8 media together with 0.25 µg/ml puromycin. Cells were selected for puromycin for 14 days and positive clones were selected by PCR. CBF$\beta$-MYH11 iPSCs were routinely maintained in E8 media (Life technologies) on vitronectin coated plates.

**Granulocytic, monocytic, erythrocytic and megakaryocytic differentiation**

For granulocytic and monocytic differentiation, CBF$\beta$-MYH11 iPSCs were dissociated using accutase and resuspended in E8 media supplemented with rock inhibitor (10 µg/ml). Cells were seeded at a density on Geltrex (Life Technologies) coated six-well plate, so that only 4-5 colonies emerge in each well. Cells were maintained in E8 media until individual colonies grew up to approximately 500 mm in diameter. E8 media was then replaced by stemline media supplemented with 1% Pen/Strep, 1:100 ITS and cytokines (20 ng/ml BMP4, 40 ng/ml VEGF and 5 ng/ml bFGF). This day was considered as day 0 of differentiation and the medium was refreshed after 3 days. On day 6, the cytokines were again replaced with a specific cytokine cocktail for monocyte (50 ng/ml SCF, 50 ng/ml FLT3, 50 ng/ml IL-3, 50 ng/ml M-CSF and 10 ng/ml TPO)^5^, neutrophil (50 ng/ml SCF, 50 ng/ml IL-3, 50 ng/ml G-CSF and 5 ng/ml TPO)^6^, erythrocyte (100x ITS, 5 U/ml EPO, 50 ng/ml hSCF, 10 ng/ml TPO, 50 ng/ml IL-6, 50 ng/ml IL-3, 1% Pen/strep)^7^ or megakaryocyte (10 ng/ml VEGF, 20 ng/ml BMP4, 1 ng/ml IL-3, 10 ng/ml IL-6, 50 ng/ml TPO, 50 ng/ml hSCF, 100x ITS, 1% Pen/strep and 10 ng/ml IL-1$\beta$)^7^ differentiation and the medium was changed every 3-4 days. Dox (14 ng) was added on the 6th day of differentiation and cells were kept continuously in dox until analyzed by flow analysis.

**Flow cytometric analysis**

Suspension cells were collected and washed in PBA buffer (1% BSA in PBS) and pre-incubated in 2% human serum to inhibit unspecific antibody binding. The cells were stained with monocyte (CD34/CD45, CD14/CD16), neutrophil (CD34/CD45, CD15/CD16) or megakaryocyte/Erythrocyte (CD41a/CD34, CD41a/CD42b, CD41a/CD235a) antibody cocktail. After staining, the cells were fixed in 1% paraformaldehyde (PFA) and analyzed on the FACS Calibur within 3 days. The data was collected and analyzed by the FlowJo software.

**Cytospin**

For morphological analysis, 5$\times$10^4^ cells were spinned for 10 minutes at 800g on a glass slide and air-dried for at least one hour at room temperature. Cells were fixed and stained for 5 minutes with May-Grünwald and 15 minutes with Giemsa staining. The slides were washed and mounted with Permount solution. Images were taken using the Zeiss Axio Scope A1 microscope with the 40$\times$ objective and analyzed using the AxioVision LE64 software (Zeiss).

**Nuclear extract preparation and pull down**

Nuclear extracts of control and CM knockdown cells (72 h sh-RNA induction) were prepared as previously described^8^, snap-frozen in liquid nitrogen and stored at -80$℃$. For pull-down, bait (containing the RUNX1 motif: **Biotin F-**AACCAAGGGCTGCAGTGGCTTCTGTGGTTTTCCCGAGACGCAGTGGAGCC) oligonucleotides were generated by annealing 4 nM of sense and 8 nM antisense strands in a total volume of 160 μl. The sense strand of bait was biotinylated at the 5’ end for coupling to streptavidin sepharose beads. 85 μl of streptavidin sepharose slurry (GE Healthcare, 17511301) was washed with DB buffer (20 mM Tris-HCl, pH 8.0, 2 M NaCl, 0.5 mM EDTA, 0.03% NP-40). 160 μl of annealed bait was added to the washed sepharose beads in a total volume of 360 μl of DB buffer and incubated for 1 hr at RT on rotation wheel. After coupling, the beads were washed two times with DBB, two times in PB buffer, resuspended in 320 µl of PBB and divided in two tubes. 1mg of nuclear lysate (ME-1 control or CBF$\beta$-MYH11 knockdown) was added to beads in a total volume of 600 μl PB buffer (150 mM NaCl, 50 mMTris/HCl pH 8.0, 10 mM MgCl2, 0.5% NP-40, Complete Protease Inhibitor-EDTA [Roche]) supplemented with 10 μg of poly dIdC and incubated for 90 minutes at 4$℃$ on a rotation wheel. The beads were then washed two times with PBB and two times with wash buffer (150 mM NaCl, 50 mM Tris/HCl pH 8.0) to remove the detergent present in the PBB. Beads were resuspended in 50 μl of urea sol (2 M urea, 50 mM HEPES pH 8.0, 10 mM DTT) and incubated for 30 min at RT with shaking. Subsequently, 50 mM IAA was added for alkylation and incubated in dark for 20 min at RT with shaking. Proteins were digested on-bead by adding 0.4 µg of a trypsin/LysC mix (Promega) and incubated for 1 h at 25$℃$ in thermoshaker. Beads were collected by centrifugation and supernatant was transferred to the collection tube. Beads were washed with 50 μl of 2 M urea in HEPES, centrifuged and the supernatant was transferred to the same collection tube. Digestion was continued at 25$℃$ overnight at RT. Next day samples were acidified by adding 10 µL of TFA, purified on StageTips^9^ and samples were dimethyl labeled.

**Dimethyl labeling and mass spectrometry**

For dimethyl labeling digested samples were eluted in 200 µl of 60% ACN and then reduced in volume by speedvac to 100 µl and divided in two tubes. 50 µl 200mM TEAB was added and dimethyl labeling was performed as described in original dimethyl protocol^10^. A standard setup in pull-down with dimethyl labeling consists of two experiments, forward and reverse. In the forward experiment pull-down proteins from control lysate is incubated with light and CM knockdown lysate with the heavy isotope. In the reverse experiment, pull down proteins from control lysate is incubated with heavy and CM knockdown lysate with light isotope. Four microliters of 4% CH2O (light) or CD2O (heavy) together with 4 µL of 0.6 M NaBH3CN was incubated with the sample for 1 h at room temperature, with shaking. The reaction was stopped by adding 16 µL of 1% NH3. Corresponding light and heavy labeled samples were merged, acidified with 5 µL of TFA and purified by stage tip.

Mass spectrometry operation and raw data analyses were conducted as described for standard dimethyl pull down^11^, in which protein group files were processed and scatter plots were generated in Perseus software.

**Primers and antibodies used in this study**

**RT-qPCR**

| **Primers** | **Forward** | **Reverse** |
| --- | --- | --- |
| CBF$\beta$-MYH11 cDNA | AAGACTGGATGGTATGGGCTGT | CAGGGCCCGCTTGGA |
| CBF$\beta$ cDNA | CTTAGAAAGAGAAGCAGGCAAGG | AACTCCAGACAGCCCATACCA |
| GAPDH cDNA | GAAGGTGAAGGTCGGAGTC | GAAGATGGTGATGGGATTTC |

**ChIP-seq antibodies**

| **Antibody^(ref)^** | **Catalogue no** | **Company** |
| --- | --- | --- |
| CBF$\beta$-sc^12, 13^ | sc-20693 | Santa Cruz |
| CBF$\beta$-A1329^13, 14^ | na | Diagenode |
| MYH11-Novus^13^ | 21370002 | Novus Biologicals |
| MYH11-A1379^13^ | na | Diagenode |
| RUNX1^12-16^ | ab23980 | Abcam |
| GATA2^13, 16^ | sc-9008 | Santa Cruz |
| H3K27ac^3^ | C15410196 | Diagenode |

**References**

1. Hansen M, Varga E, Wust T, Brouwer N, Beauchemin H, Mellink C*, et al.* Generation and characterization of human iPSC line MML-6838-Cl2 from mobilized peripheral blood derived megakaryoblasts. *Stem Cell Res* 2017 Jan; **18:** 26-28.

2. Mali P, Yang L, Esvelt KM, Aach J, Guell M, DiCarlo JE*, et al.* RNA-guided human genome engineering via Cas9. *Science* 2013 Feb 15; **339**(6121)**:** 823-826.

3. Mandoli A, Singh AA, Prange KHM, Tijchon E, Oerlemans M, Dirks R*, et al.* The Hematopoietic Transcription Factors RUNX1 and ERG Prevent AML1-ETO Oncogene Overexpression and Onset of the Apoptosis Program in t(8;21) AMLs. *Cell Rep* 2016 Nov 15; **17**(8)**:** 2087-2100.

4. Qian K, Huang CT, Chen H, Blackbourn LWt, Chen Y, Cao J*, et al.* A simple and efficient system for regulating gene expression in human pluripotent stem cells and derivatives. *Stem Cells* 2014 May; **32**(5)**:** 1230-1238.

5. Niwa A, Heike T, Umeda K, Oshima K, Kato I, Sakai H*, et al.* A novel serum-free monolayer culture for orderly hematopoietic differentiation of human pluripotent cells via mesodermal progenitors. *PLoS One* 2011; **6**(7)**:** e22261.

6. Morishima T, Watanabe K, Niwa A, Hirai H, Saida S, Tanaka T*, et al.* Genetic correction of HAX1 in induced pluripotent stem cells from a patient with severe congenital neutropenia improves defective granulopoiesis. *Haematologica* 2014 Jan; **99**(1)**:** 19-27.

7. Hansen M, Varga E, Aarts C, Wust T, Kuijpers T, von Lindern M*, et al.* Efficient production of erythroid, megakaryocytic and myeloid cells, using single cell-derived iPSC colony differentiation. *Stem Cell Res* 2018 May; **29:** 232-244.

8. Spruijt CG, Gnerlich F, Smits AH, Pfaffeneder T, Jansen PW, Bauer C*, et al.* Dynamic readers for 5-(hydroxy)methylcytosine and its oxidized derivatives. *Cell* 2013 Feb 28; **152**(5)**:** 1146-1159.

9. Rappsilber J, Mann M, Ishihama Y. Protocol for micro-purification, enrichment, pre-fractionation and storage of peptides for proteomics using StageTips. *Nat Protoc* 2007; **2**(8)**:** 1896-1906.

10. Boersema PJ, Raijmakers R, Lemeer S, Mohammed S, Heck AJ. Multiplex peptide stable isotope dimethyl labeling for quantitative proteomics. *Nat Protoc* 2009; **4**(4)**:** 484-494.

11. Hubner NC, Nguyen LN, Hornig NC, Stunnenberg HG. A quantitative proteomics tool to identify DNA-protein interactions in primary cells or blood. *J Proteome Res* 2015 Feb 6; **14**(2)**:** 1315-1329.

12. Yu M, Mazor T, Huang H, Huang HT, Kathrein KL, Woo AJ*, et al.* Direct recruitment of polycomb repressive complex 1 to chromatin by core binding transcription factors. *Mol Cell* 2012 Feb 10; **45**(3)**:** 330-343.

13. Mandoli A, Singh AA, Jansen PW, Wierenga AT, Riahi H, Franci G*, et al.* CBFB-MYH11/RUNX1 together with a compendium of hematopoietic regulators, chromatin modifiers and basal transcription factors occupies self-renewal genes in inv(16) acute myeloid leukemia. *Leukemia* 2014 Apr; **28**(4)**:** 770-778.

14. Martens JH, Mandoli A, Simmer F, Wierenga BJ, Saeed S, Singh AA*, et al.* ERG and FLI1 binding sites demarcate targets for aberrant epigenetic regulation by AML1-ETO in acute myeloid leukemia. *Blood* 2012 Nov 8; **120**(19)**:** 4038-4048.

15. Tijssen MR, Cvejic A, Joshi A, Hannah RL, Ferreira R, Forrai A*, et al.* Genome-wide analysis of simultaneous GATA1/2, RUNX1, FLI1, and SCL binding in megakaryocytes identifies hematopoietic regulators. *Dev Cell* 2011 May 17; **20**(5)**:** 597-609.

16. Wilson NK, Foster SD, Wang X, Knezevic K, Schutte J, Kaimakis P*, et al.* Combinatorial transcriptional control in blood stem/progenitor cells: genome-wide analysis of ten major transcriptional regulators. *Cell Stem Cell* 2010 Oct 8; **7**(4)**:** 532-544.
